# The Development of Crassulacean Acid Metabolism (CAM) Photosynthesis in Cotyledons of the C_4_ Species, *Portulaca grandiflora* (Portulacaceae)

**DOI:** 10.3390/plants9010055

**Published:** 2020-01-02

**Authors:** Lonnie J. Guralnick, Kate E. Gilbert, Diana Denio, Nicholas Antico

**Affiliations:** Department of Biology, Roger Williams University, One Old Ferry Rd., Bristol, RI 02809, USA; kgilbert721@g.rwu.edu (K.E.G.); ddenio335@g.rwu.edu (D.D.); nantico949@g.rwu.edu (N.A.)

**Keywords:** *Portulaca grandiflora*, C_4_ photosynthesis, Crassulacean acid metabolism (CAM), evolution, development, PEP carboxylase, Portulacaceae, glycine decarboxylase

## Abstract

*Portulaca grandiflora* simultaneously utilizes both the C_4_ and Crassulacean acid metabolism (CAM) photosynthetic pathways. Our goal was to determine whether CAM developed and was functional simultaneously with the C_4_ pathway in cotyledons of *P. grandiflora*. We studied during development whether CAM would be induced with water stress by monitoring the enzyme activity, leaf structure, JO_2_ (rate of O_2_ evolution calculated by fluorescence analysis), and the changes in titratable acidity of 10 and 25 days old cotyledons. In the 10 days old cotyledons, C_4_ and CAM anatomy were evident within the leaf tissue. The cotyledons showed high titratable acid levels but a small CAM induction. In the 25 days old cotyledons, there was a significant acid fluctuation under 7 days of water stress. The overall enzyme activity was reduced in the 10 days old plants, while in the 25 days old plants CAM activity increased under water-stressed conditions. In addition to CAM, the research showed the presence of glycine decarboxylase in the CAM tissue. Thus, it appears both pathways develop simultaneously in the cotyledons but the CAM pathway, due to anatomical constraints, may be slower to develop than the C_4_ pathway. Cotyledons showed the ancestral Atriplicoid leaf anatomy, which leads to the question: Could a CAM cell be the precursor to the C_4_ pathway? Further study of this may lead to understanding into the evolution of C_4_ photosynthesis in the *Portulaca.*

## 1. Introduction

CO_2_ concentrating mechanisms have evolved in terrestrial plants in response to changing environmental conditions. Two different mechanisms have evolved that involve a similar suite of enzymes utilized in a different fashion to overcome photorespiration and increased water loss. Photorespiration increases when CO_2_ becomes limited under high light intensities and high evaporative demand resulting in increased transpirational water loss [1]. The C_4_ pathway overcomes these limitations with the CO_2_ being initially captured as HCO_3_^-^ by phosphoenolpyruvate carboxylase (PEPCase) and then fixed via the C_3_ pathway by Rubisco. C_4_ plants have a spatial separation of the C_4_ and C_3_ pathways occurring within two different cell types in the leaf. The C_4_ pathway, located in the palisade mesophyll cells, is radially arranged around the C_3_ pathway located in the bundle sheath cells, which surround the vascular tissue. This is typically referred to as Kranz anatomy [1,2]. Research by Voznesenskaya et al. [3,4] has shown that the Kranz anatomy is not essential for terrestrial C_4_ photosynthesis to occur but it can occur in a single cell with a spatial separation of the C_4_ and C_3_ pathways within a single chlorenchyma cell. The C_4_ pathway concentrates CO_2_ at the site of Rubisco and helps to suppress photorespiration in the bundle sheath cells. The C_4_ pathway has been found in approximately 19 plant families and over 8000 species [1] and has evolved independently many times.

Crassulacean acid metabolism (CAM) is a metabolic and anatomical adaptation that is characterized by net nocturnal carbon dioxide uptake with a temporal separation of the C_4_ and C_3_ pathway resulting in decreased transpiration rates and water loss [5,6]. The CO_2_ is similarly fixed (as in C_4_ plants) by PEPCase, converted to malate and stored as malic acid in the large central vacuole during the night period. In the subsequent light period, the malate is transported out of the vacuole and then decarboxylated to release CO_2_ for utilization by Rubisco in the C_3_ cycle. CAM plants typically have a mesophyll anatomy with primarily spongy parenchyma cells with a large central vacuole, which has the ability to store the increasing accumulation of malic acid during the nocturnal period [7]. CAM plants anatomically have very low mesophyll airspace, so when the stomata are closed the CO_2_ concentrations inside the leaf can reach very high levels to suppress photorespiration [6,7]. CAM has evolved in at least 34 different plant families including 6 aquatic families and over 20,000 species [1,8].

CAM and C_4_ photosynthesis have evolved independently multiple times in many different plant families. One might hypothesize both CAM and C_4_ could have evolved in the same plant families numerous times due to the similarity of the enzymes involved in the pathways. The two pathways have evolved in the same family four times (Aizoaceae, Asteraceae, Euphorbiaceae, Portulacaceae). This raises an interesting question about why the C_4_ and CAM pathways have only concurrently evolved in these four families. The original circumscription of the Portulacaceae describes the family as containing approximately 29 genera [9]. Guralnick and Jackson [10] have reported the evolution and distribution of C_4_ and CAM photosynthesis found in this family. It has been observed that some members are C_3_ plants, while others are C_3_ plants with attributes of CAM. Others are C_4_ plants with some CAM characteristics, and the more advanced species are facultative CAM plants [10,11,12].

The genus *Portulaca* is known to have the only C_4_ photosynthetic members of the family despite previous reports to the contrary [11]. This revision reveals the genus *Portulaca* (Portulacaceae) contains the only known C_4_ members that are capable of CAM photosynthesis [13]. *Portulaca spp.* tend to inhabit environments with high light intensities, which periodically become dry. The genus *Portulaca* has succulent stems and leaves and because of the high degree of succulence, there have been reports in the literature that members of the *Portulaca* show a diurnal acid fluctuation characteristic of CAM species. Koch and Kennedy [14,15] showed *Portulaca oleracea* having a diurnal acid fluctuation in both the stems and leaves. They also measured low levels of net nighttime CO_2_ uptake. Research done by Guralnick and Jackson [10] showed that *Portulaca mundula*, *P. pilosa*, and *P. oleracea* exhibited high acid levels and diurnal acid fluctuations indicative of CAM photosynthesis. Kraybill and Martin [16] showed both *P. oleracea* and *P. grandiflora* both undergo CAM cycling with little or no nocturnal CO_2_ uptake. Mazen [17] indicated that under water stress conditions that *P. oleracea* had increased levels of PEP (phosphoenolpyruvate) carboxylase protein. Further research showed the genus contains a C_3_-C_4_ intermediate species, *Portulaca cryptopetala*, in which recent work showed *P. cryptopetala* induced CAM under water stress conditions [2,18]. Winter et al. [18] extended the findings to additional species in the *Portulaca* and also consider them to be facultative CAM species.

*Portulaca grandiflora* is a small herbaceous annual utilizing the C_4_ photosynthetic pathway. *P. grandiflora* has small succulent leaves with a Pilosoid-type Kranz leaf anatomy where the C_4_ tissue in the succulent leaves surround the large water storage tissue [2,19]. *P. grandiflora* is known to maintain high organic acid levels and shows a large diurnal acid fluctuation when water stressed, typical of CAM species [20]. Research has indicated the increase in CAM of this species occurs in the water storage portion of the leaf and the stem during water stressed conditions [20]. *Portulaca grandiflora* is unique because it has both C_4_ and CAM photosynthetic pathways operating simultaneously in the leaf tissue [20]. This situation is unique due to the proposed incompatibility of both pathways to operate in the same leaf [21]. Phylogenetic analysis has indicated the genus *Portulaca* evolved CAM from a C_3_ ancestor prior to the appearance of the C_4_ pathway [10]. The objective of this study was to study cotyledon leaf tissue to determine if both the CAM and C_4_ pathways were developing and operating simultaneously. CAM induction in developing cotyledons was monitored by withholding water for 3 and 7 days. An understanding of the developmental process of these pathways will aid in clarifying the evolutionary origins of the CAM and C_4_ pathways in the Portulacaceae.

## 2. Results

### 2.1. Titratable Acidity

**Figure 1:**
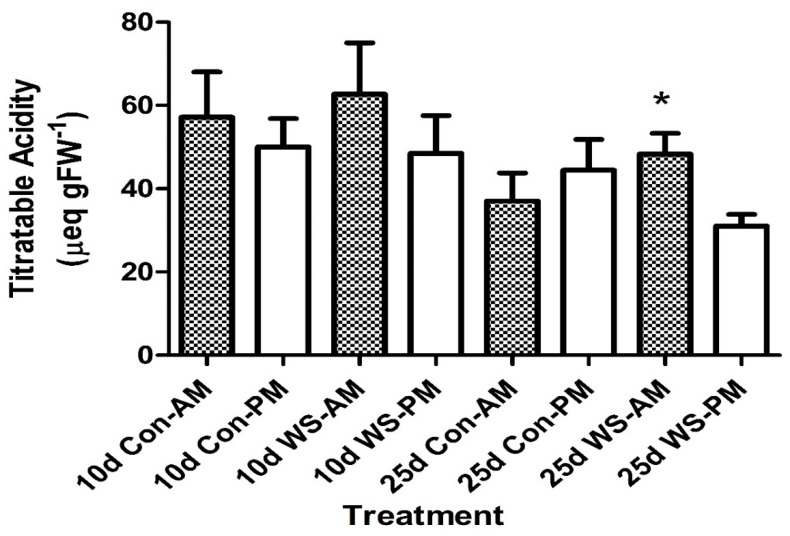
Titratable acidity of *Portulaca grandiflora* in 10 days and 25 days old cotyledons under control and 3 days water-stress conditions. Bars represent the means (SEM). For 10 days old, N = 9–11 leaves per treatment; 25 days old, N = 8–13 leaves per treatment; and * indicates a significant difference between a.m. and p.m. acid levels for 25 days old treatment. Con = Control; WS = Water Stress for all figures.

**Figure 2:**
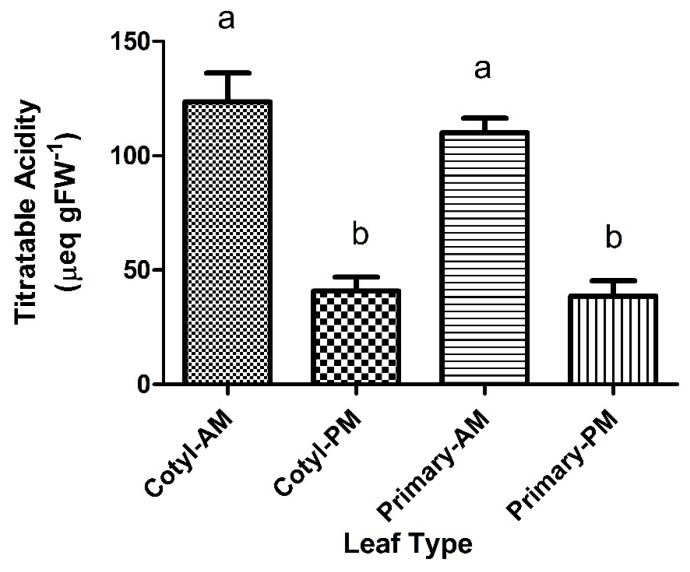
Diurnal titratable acidity levels in cotyledons and primary leaves of *P. grandiflora* after 7 days of water stress. Bars represent the means (SEM). Bars with different letters are significantly different (*p* < 0.05, N = 4).

Titratable acidity levels for 10 days old cotyledons were at approximately 50–60 µeq gFW^−1^ (FW = Fresh Weight; Figure 1). At 10 days, the control groups and water-stressed leaves showed a slight acid fluctuation of 10–20 µeq gFW^−1^ from a.m. to p.m. (Figure 1). There was no significant difference between a.m. and p.m. acid levels. At 20–25 days old, cotyledons, under control conditions, there was no acid fluctuation observed from a.m. to p.m. levels. Under water stress, cotyledons showed a small significant titratable acid fluctuation from the morning to the evening (Figure 1). Continued water stress to 7 days of the 20–25 days old cotyledons induced a large and significant acid fluctuation of 83 µeq gFW^−1^ in both the cotyledons and primary leaves of *P. grandiflora* (Figure 2). The a.m. acid levels had increased to more than double the control cotyledons.

### 2.2. Enzyme Activity: PEP Carboxylase and NADP-ME

**Figure 3:**
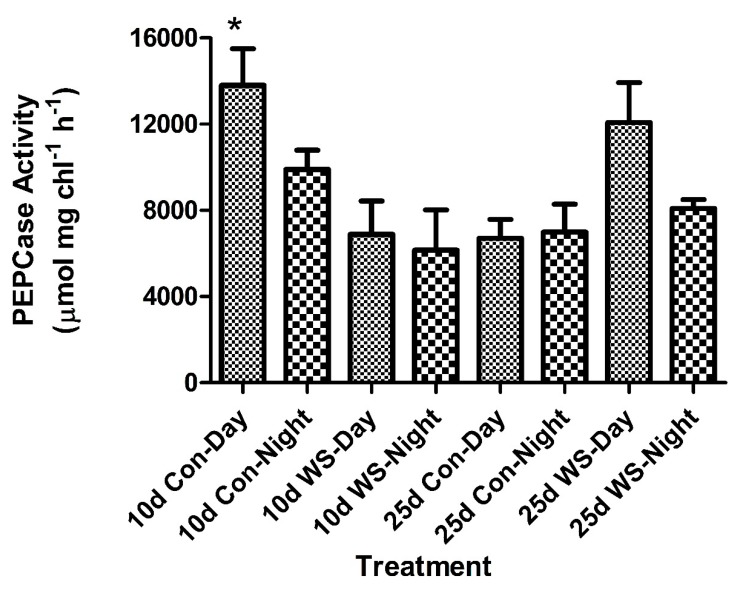
PEP carboxylase activity of *P. grandiflora* in 10 days and 25 days old cotyledons under control and 3 days water-stress conditions. Bars represent the means (SEM); * = 10 days Con-Day significantly different from 10 days WS-Day (*p* < 0.05, N = 8–12); 25 days old cotyledons N = 21–25 control plants, N = 8–11 water-stress plants.

Ten day old cotyledons showed PEPCase activity higher during the day than at night (Figure 3). Water stress lowered the activity of PEPCase in the 10 days old cotyledons during the day and night. There was a significant difference in daytime activity between the control and water stress at 10 days. At 25 days old, the activity remained high during the day but lower at midnight (Figure 3). In the 25 days old cotyledons, water stress had less of an effect on the overall activity of PEPCase. The PEPCase activity during the day increased in the water-stress plants compared to the control plants (Figure 3). There was no significant differences in PEPCase activity in the 25 days old cotyledons (Figure 3).

**Figure 4:**
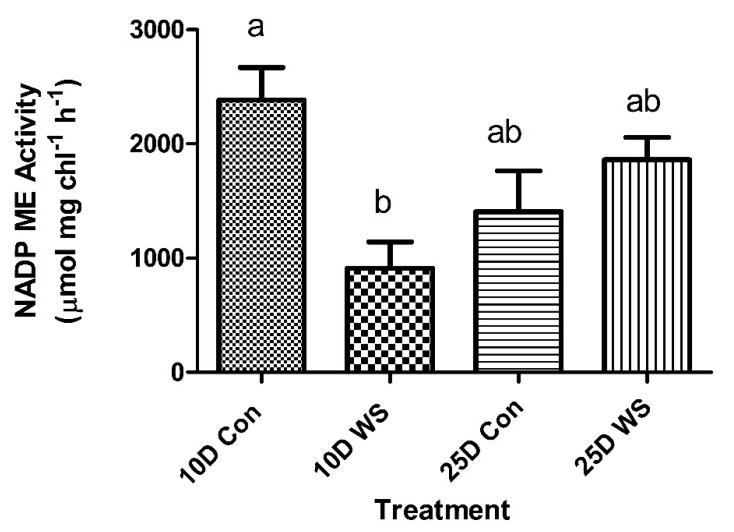
NADP-ME activity of *P. grandiflora* in 10 days and 25 days old cotyledons under control and 3 days water-stress conditions. Bars represent the means (SEM). N = 6–13. Bars with different letters are significantly different (*p* < 0.05).

The decarboxylase, NADP-Malic Enzyme (ME), showed high daytime activity in both the 10 days and 25 days old control cotyledons (Figure 4). Water stress significantly lowered the NADP-ME activity at 10 days, while at 25 days there was no significant change in the level of activity between the control and water-stressed plants.

### 2.3. Electron Transport Rate/JO_2_

**Figure 5:**
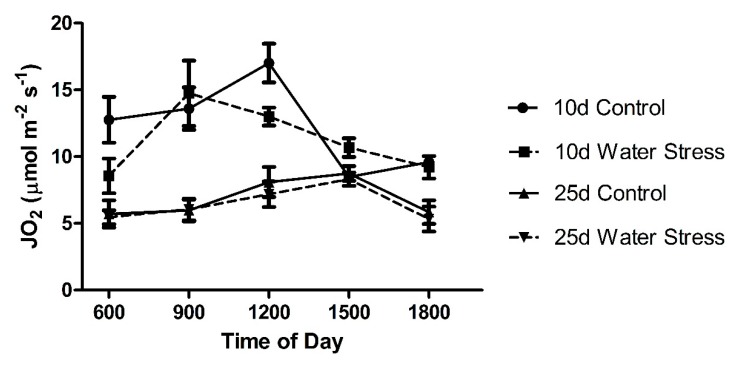
JO_2_ rates of leaf samples for 10 days and 25 days control and water-stress treatments. Error bars represent one standard error of the mean. N = 12–14 leaves per time point.

The JO_2_ rates showed no differences between control and water-stressed plants in 10 days old and 25 days old cotyledons (Figure 5). The JO_2_ rates were lower in 25 days old cotyledons than in the 10 days old cotyledons.

The gross rate of photosynthesis (JO_2_) showed a peak at midday for the well-watered 10 days old cotyledons while the stressed cotyledons showed a peak earlier in the morning (Figure 5). In the 25 days old cotyledons, the JO_2_ calculated rates were relatively constant over the course of the light period. There was no difference between the control and water-stressed plants in the JO_2_ rates (Figure 5).

### 2.4. Leaf Anatomy

**Figure 6:**
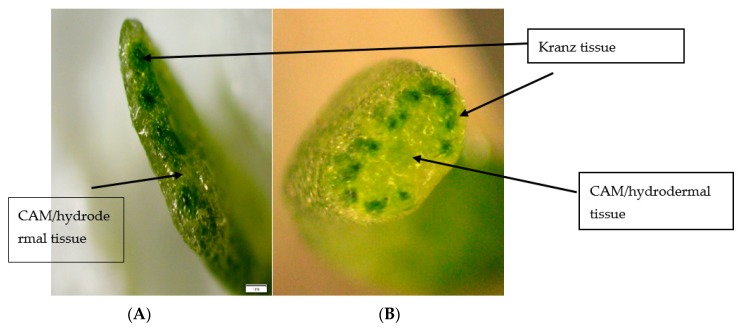
Cross section of 1.7× magnification of (**A**) ~20 days old cotyledon and (**B**) mature leaf of *P. grandiflora*. The dark green bundles within the leaf tissue are the C_4_ Kranz anatomy with high levels of chlorophyll. The lighter areas are the CAM/hydrodermal tissue.

**Figure 7:**
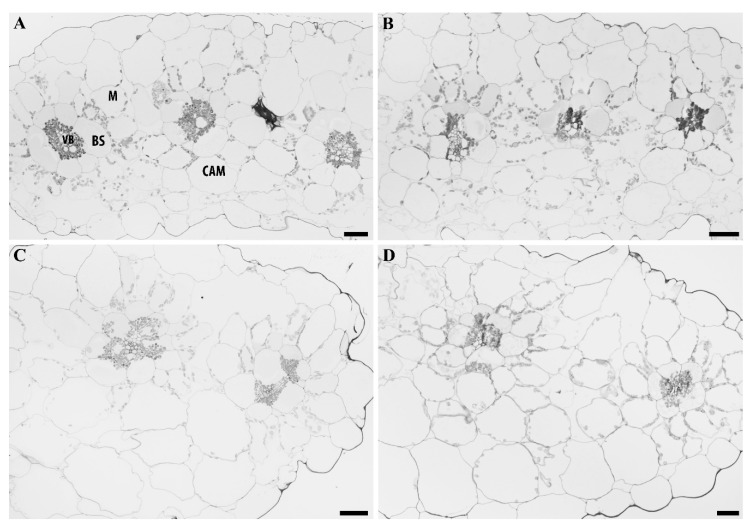
Light microscope images of cotyledons of *P. grandiflora* at low magnification, (**A**) 10 days control, (**B**) 10 days water stress, (**C**) 25 days control, (**D**) 25 days water stress. Bars = 50 µm, VB = vascular bundle, BS = bundle sheath, M = mesophyll, CAM = hypodermal tissue.

*Portulaca grandiflora* cotyledons were dissected and photographed to display the internal gross anatomy of the tissue (Figure 6). The sections illustrate the Atriplicoid Kranz anatomy in the cotyledon (Figure 6A), and its linear arrangement compared to a mature leaf where the Kranz anatomy shows a radial arrangement (Figure 6B). A low-magnification view of a cotyledon leaf tissue showed a well-developed Atriplicoid Kranz anatomy arranged in a linear fashion (Figure 7). Mesophyll cells were well developed with chloroplast along the cell wall (Figure 7A). The bundle sheath cells were also well developed with chloroplasts located along the periphery of the inner cell wall close to the vascular bundles (Figure 7A). The CAM/hydrodermal tissue showed fewer chloroplasts than the C_4_ tissue (Figure 7A). At 25 days, the Kranz anatomy was very well developed and the vascular tissue was more prominent. Under water-stress conditions, the CAM/hypodermal tissue at 10 days showed signs of water loss compared to the control cotyledon tissue. The cells appeared to have some shriveling and a more irregular shape. At 25 days, there were less noticeable changes under water-stress conditions in the succulent mesophyll and hypodermal tissue (Figure 7).

**Figure 8:**
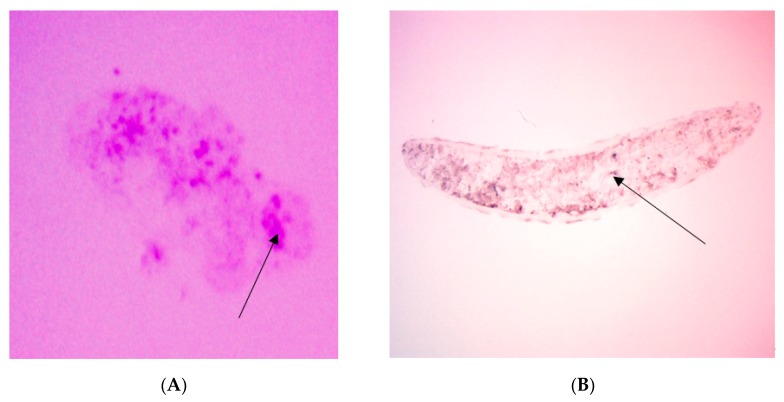
Glycine decarboxylase (GDC) protein presence by tissue printing. (**A**) Cotyledon leaf samples were sectioned and photographed, then printed onto nitrocellulose, incubated with GDC antibody, and visulaized. The arrow points to the bundle sheath tissue which showed more GDC protein. The GDC protein was also found present in the surroounding succulent CAM tissue. (**B**) *Portulacaria afra*, a facultative CAM species, leaf tissue print with the arrow indicating GDC antibody found throughout the spongy parenchyma tissue.

The sections illustrate the Atriplicoid Kranz anatomy in its linear arrangement compared to a mature leaf where the Kranz anatomy shows a radial arrangement (Figure 6B). We performed tissue prints of cotyledons to determine the presence of glycine decarboxylase (GDC, a mitochondrial marker for the photorespiratory pathway). GDC was found to be present in the CAM/hydrodermal tissue of *P. grandiflora* cotyledons (Figure 8B). In mature leaves of *P. grandiflora*, GDC was also located in the inner CAM/hydrodermal tissue (data not shown). For comparison, *Portulacaria afra*, a facultative CAM species, showed the presence of GDC throughout the spongy parenchyma mesophyll tissue (8b).

## 3. Discussion

Guralnick and Jackson [10] previously suggested the CAM pathway appeared evolutionarily first in the leaf and the C_4_ photosynthetic pathway overlaid the CAM tissue. These conclusions were supported by research which showed the CAM PEPCase gene was more primitive to the C_4_ derived PEPCase gene [22]. Sage [21] described the leaf anatomy found in the *Portulaca* as being unique because of the apparent incompatibility of the photosynthetic pathways. The genus *Portulaca* shows nearly all members display C_4_ photosynthesis with attributes of the CAM pathway in the same leaf tissue and are considered facultative CAM species [18]. Previous research showed the CAM pathway aids in the retention of water for maintenance of the C_4_ pathway and the recycling of carbon to the mesophyll and bundle sheath cells [20]. This cooperation between the tissues may be the result of the close proximity of the tissues to each other because previous work has proposed that the tissues are functioning independently of each other [20].

### 3.1. C_4_ Development

Research has been performed on the physiological and structural development of C_4_ photosynthesis in cotyledons of the *Portulaca* but little of CAM development in these C_4_ species [23]. The research presented here indicated it was apparent that the C_4_ pathway was well developed in 10 days old cotyledons based on the anatomy and physiology. The leaf anatomy of the cotyledons showed a well-developed Kranz C_4_ anatomy as found previously in the genus *Portulaca* [23]. *P. grandiflora* cotyledons displayed an Atriplicoid-type anatomy where the vascular bundles are in one plane of the leaf. The Atriplicoid Kranz anatomy showed CAM tissues around the periphery of the leaf surrounding the Kranz bundles. This differs from the Pilosoid Kranz anatomy found in mature leaves of *P. grandiflora* with the C_4_ tissue in a ring surrounding the CAM water storage tissue [2,20].

The enzymes of the C_4_ pathway were fully functional at 10 days, as shown by a high activity of PEPCase and NADP-ME. This was comparable to the conclusions reached by Dengler et al. [24] for *Atirplex rosea*, which showed accumulation of PEPCase was detected 2–4 days after leaf development and expansion. The expression was limited to mesophyll tissue adjacent to the bundle sheath tissue. The JO_2_ studies in this study showed high rates of photosynthesis indicative of C_4_ activity and were comparable to the rates previously measured in mature leaf tissue [20]. The C_4_ pathway is the primary CO_2_ acquiring pathway in this genus and it was predictable the C_4_ pathway developed quickly for seedling establishment.

### 3.2. CAM Development

The development of CAM, which can be measured by titratable acidity levels and diurnal acid fluctuations, showed a slower development in cotyledons from that of the C_4_ pathway. The study of CAM development showed comparable titratable acidity levels but slightly lower and comparable to mature leaf tissues under control conditions [20]. One can measure significant total titratable acidity levels in the cotyledons at 10 and 25 days. The acid levels found in the 10 days old cotyledons of *P. grandiflora* are similar but larger than those found in well-watered cotyledons of *Mesembryanthemum crystallinum*, a facultative CAM species [25], and higher than the acid levels in the CAM-cycling species, *Lewisia cotyledon* [26]. The acid levels of *P. grandiflora* were lower than those found in a number of columnar cactus seedlings at 1 day and 7 days old [27]. The acid levels were lower than those for *Opuntia elatior* at 23 days old cotyledons [28]. However, these cactus species primarily utilize the CAM pathway for CO_2_ uptake, even in cotyledons. The cotyledon anatomy in cactus has succulent anatomy with a spongy parenchyma tissue with little airspace, which is more conducive to CAM photosynthesis [29,30]. The results in the present study indicated *P. grandiflora* cotyledons showed similar total titratable acid levels comparable to other CAM species.

We investigated the potential for CAM activity by water stressing the cotyledons for three and seven days. Our results differed with the age of the cotyledons and duration of the drought. Ten days old cotyledons showed small acid fluctuations indicative of CAM activity after three days of water stress. The 25 days old water-stressed cotyledons showed a small acid fluctuation compared to control cotyledons which showed no acid fluctuation. Continuation of water stress for seven days induced a very large and significant CAM acid fluctuation in both cotyledons and primary leaf tissue. The acid fluctuation measured in the cotyledons was comparable to acid levels in mature leaf tissue. This indicated an induction of CAM similar to previous research on *P. grandiflora* [20] and was indicative of CAM activity.

In addition, enzyme activity, as measured by nocturnal PEPCase activity, was quite high and comparable to C_4_ rates of PEPCase measured during the day. Water stress only had a slight effect on the overall activity of PEPCase. NADP-ME was affected more by water stressed conditions and this result had been observed previously in *P. grandiflora* [20]. Water stress in other facultative CAM plants, such as *Portulacaria afra*, show the decarboxylase enzymes are more affected than PEPCase [31]. The enzyme activities of PEPCase and NADP-ME reported here in cotyledons of *P. grandiflora* were much higher than those reported for cotyledons of *Salsola* spp. [32] and for mature leaves of *P. grandiflora* [20] and may be related to the lower chlorophyll concentrations.

The physiological and anatomical changes differed with the age. At 10 days, water stressing the cotyledons induced a small acid fluctuation and it lowered both the day and night activity of PEPCase. NADP-ME activity also decreased in 10 days old cotyledons. This indicated a decrease in C_4_ and CAM activity. It appears that NADP-ME is more sensitive to water stress than Rubisco and PEPCase. Anatomically, the mesophyll CAM tissue showed some shrinkage, which may be due to a redistribution of water from the CAM cells to the C_4_ metabolic cells. This has been observed in mature leaves of *P. grandiflora* during a 10 days drought [20]. The sensitivity of the enzymes due to drought at 10 days may be due more in part to structural development of the CAM tissue and water storage at this stage. The enzymatic activity appeared earlier prior to full anatomical development of the CAM tissue. This is similar to the development of C_4_ photosynthesis in *A. rosea*, which showed differential enzyme expression of the C_4_ photosynthetic enzymes by 4 days [24].

By 20–25 days old, the response to water stress and CAM activity was similar to the 10 days old cotyledons. There was a similar induction of an acid fluctuation in water-stressed plants. Seven days of water stress induced a much larger acid fluctuation indicative of CAM activity and greater than observed after three days of water stress. This acid fluctuation was similar to acid fluctuations in other CAM-cycling species [10,20]. The results indicated an induction of CAM activity similar to control plants of *Lewisia cotyledon* [26]. Induction of water stress did not lower PEPCase activity in 25 days old cotyledons compared to 10 days old cotyledons. NADP-ME did not show a decrease in activity due to water stress and showed a slight increase in activity, which may be due to increased CAM activity of the leaf. The enzyme activity indicated a maintenance of the C_4_ pathway in the 25 days old cotyledons. This was supported by the JO_2_ data, which showed no changes in activity during three days of drought. Structurally, the CAM/hydrodermal tissue was more mature in the 25 days old cotyledons and did not appear to show as much water loss as the 10 days old cotyledons. This may explain the ability of older cotyledons to maintain C_4_ activity when compared to 10 days old cotyledons. The continuation of drought showed a much stronger induction of CAM and was very similar to the response of mature leaves [20]. The CAM tissue in mature leaves recycles CO_2_, redistributes water to the C_4_ tissue to aid in the survival of the plant, and may play a similar role in cotyledons [20].

Mature leaves of *P. grandiflora* have a Pilosoid-type anatomy with a ring of bundles surrounding the water storage tissue [2,20]. *P. grandiflora* cotyledons have an Atriplicoid-type anatomy where the vascular bundles are in one plane of the leaf. This arrangement is considered ancestral to the Pilosoid arrangement with the water storage tissue found on both the abaxial and adaxial sides of the leaf [33]. Voznesenskaya et al. [2] reported on the diversity of structure in the *Portulaca* and found the arrangement of an inner water storage, the derived condition found in *P. grandiflora*, *P. pilosa*, and *P. amilis* [2], to be due to the ecological constraints of being found in more semi-arid regions than other *Portulaca* spp. However, they did not report any CAM activity in the cotyledons of the *Portulaca spp*. in their study. Further, we report the presence of glycine decarboxylase, a mitochondrial marker for the C_2_ photorespiratory pathway in the CAM tissue of cotyledons. Previous research has shown the GDC to be localized in the mitochondria of bundle sheath cells in cotyledons [2]. The presence of GDC adds another dimension to another function of CAM in *P. grandiflora*. Previous work indicated CAM tissue might transfer water from the tissue to the Kranz anatomy to help maintain C_4_ photosynthetic activity under water stress. Additionally, decarboxylation of the acid may produce CO_2_ for the adjacent mesophyll and bundle sheath tissue. We now suggest the C_2_ pathway may provide an additional source of CO_2_ for the C_4_ pathway. It was shown the photorespiratory pathway may elevate CO_2_ levels three-fold in the leaf in *Flaveria pubescens*, (a C_3_-C_4_ intermediate) [34]. The photorespiratory pathway in the CAM tissue of *P. grandiflora* may provide an additional source of CO_2_ for the C_4_ pathway. Since CAM tissue has reduced airspace, the Atriplicoid arrangement may reduce diffusion of water out of the leaf and maintain CO_2_ levels around the Kranz anatomy. This may aid in survival of the seedlings. Further work will be needed to investigate the role of photorespiration in the different Kranz types CAM tissue in *P. grandiflora*.

This is the first report of CAM activity in the cotyledons and the results indicated CAM can be induced in cotyledons of *P. grandiflora*. The development of CAM occurred after the development of the C_4_ photosynthetic pathway and may be due to more of a constraint of leaf anatomy of the CAM tissue in cotyledons. The retention of CAM in the cotyledons of *P. grandiflora* further supports the idea that CAM was an ancient pathway in the genus *Portulaca* including the retention of the C_2_ pathway. Since the cotyledons have a different type of Kranz anatomy and water storage tissue than the mature leaves in *P. grandiflora*, this leads to questions on the evolution of C_4_ and CAM within the genus *Portulaca*. Research on the origins of CAM in *Portulaca* using PEPCase have indicated the CAM-specific gene was similar in sequence in other species utilizing CAM [22]. The question that arises: Could C_4_ photosynthesis have evolved from the CAM cells? Christin et al. [22] have shown the C_4_ specific PEPCase gene appears to have evolved from a non-photosynthetic form rather than the CAM form. They also suggest the other enzymes required for C_4_ photosynthesis may not have required new genes but utilization of genes already present in the cell. Since CAM cells in the *Portulaca* retain all features of C_3_ and CAM photosynthesis including the C_2_ pathway, it maybe that novel cells types of the mesophyll and bundle sheath may have differentiated from CAM-like cells. During the slow evolution of C_4_ photosynthesis as suggested by Christin et al. [22], a change in the regulatory sequences of PEPCase and GDC would be required as the cells evolved. Current research does not support the idea but questions remain on the change from a CAM-type leaf structure evolved into a C_4_ photosynthetic leaf in *Portulaca.*

## 4. Materials and Methods

### 4.1. Plant Material

**Figure 9:**
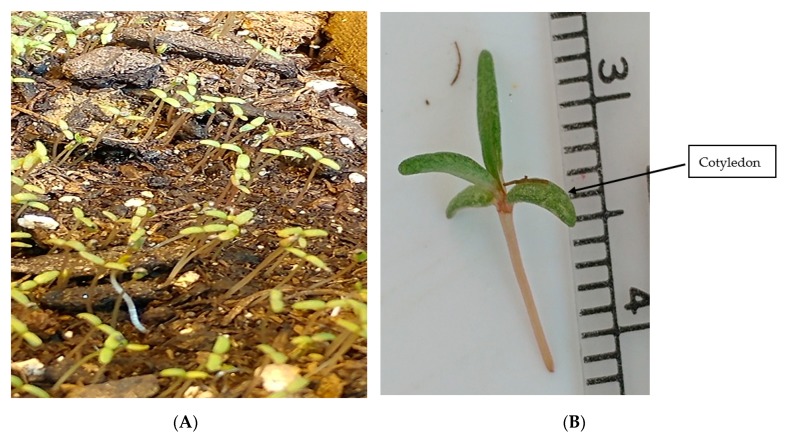
(**A**) Ten-day-old cotyledons growing flat. (**B**) Approximately 20–25 days old *P. grandiflora* seedling with cotyledons and primary leaves.

Seeds of *Portulaca grandiflora* were germinated in flats (Figure 9A,B). All plants grown were irrigated with half-strength Hoagland’s solution prior to sampling. The plants were grown under natural light conditions, supplemented with artificial light for a light intensity of 300–400 µmol m^−2^ s^−1^.

The day/night temperature in the glasshouse was 27 °C/17 °C. Seedlings were irrigated daily to maintain high water potential, and water stressed for three days prior to sampling. Experiments were performed when the leaf tissues were 10–14 days old and 22–26 days old post-germination. Leaf samples were taken during the course of the day/night cycle and frozen until assayed for titratable acidity and enzyme activity.

### 4.2. Titratable Acidity

Cotyledons were harvested in the morning and evening, and frozen (−80 °C) until assayed. Leaf samples were weighed, ground in glass-distilled water, and titrated with 0.01 N KOH to a pH 7 endpoint as described by Guralnick et al. [20]. Results were expressed as µeq gFW^−1^.

### 4.3. Enzyme Activity

Frozen leaf tissue collected midday and during the middle of the night period was utilized for phosphoenolpyruvate carboxylase (PEPCase) and NADP-malic enzyme (ME) activity. Assays of crude extracts were done in triplicate under well-watered and water-stress conditions. The samples were assayed spectrophotometrically by following the oxidation of NADH (for PEPCase) or the reduction of NADP^+^ at 340 nm as previously described by Guralnick and Ting [31]. Results were expressed as µmol mg chl^−1^ h^−1^.

### 4.4. Leaf Anatomy

Cotyledons were harvested and cut into sections. They were fixed in 1.5% glutaraldehyde in sodium cacadylate buffer followed by fixation in osmium. Sections were dehydrated in a series to 80% ethanol. The leaf material for transverse cross-section was fixed with fluoroacetic acid and then embedded in JB4 plastic resin (2-hydroxyethyl methacrylate). Sections were cut with a microtome and stained with 1% aniline B.

### 4.5. Electron Transport and JO_2_

The electron transport rate was determined using the pulse amplitude modulated fluorometer (OSP1 Fluorometer), according to Guralnick et al. [20]. Samples were taken over the course of the light period beginning at 6:00 in the greenhouse. Intact leaf tissue of well-watered and water-stressed cotyledons were measured [20]. The true rate of O_2_ evolution (JO_2_) from Photosystem II activity was calculated, according to the method of Lal and Edwards [35].

### 4.6. Tissue Printing

Cotyledons were cut with a razor and then photographed. The sections were pressed onto nitroceullusoe membranes. Protein localization of Glycine decarboxylase (GDC-H) antibodies was performed according to the method of Guralnick et al. [20]. Rabbit anti-GDC-H antibodies were purchased from Agrisera and diluted to a 1:5000 concentration. The presence of GDC was visualized using a secondary antibody linked to alkaline phosphatase and then photographed. GDC was a mitochondrial marker for the C_2_ photorespiratory pathway.

## 5. Conclusions

Research has shown the cotyledons of *Portulaca grandiflora* could be induced to perform CAM similar to mature leaves [20]. The CAM pathway appears to develop later due to anatomical but not physiological aspects of CAM. In the species, *P. grandiflora,* both pathways are present and appear to be operating independently in the cotyledons but CAM still aids the C_4_ pathway under water-stress conditions. Additionally, the C_2_ pathway may raise CO_2_ concentrations inside the cotyledons, aiding in raising the internal CO_2_ concentrations. More research will be needed to understand the evolution of CAM and C_4_ photosynthesis in the genus *Portulaca*.
